# *Kiss1* mutant placentas show normal structure and function in the mouse

**DOI:** 10.1016/j.placenta.2014.10.016

**Published:** 2015-01

**Authors:** A.M. Herreboudt, V.R.L. Kyle, J. Lawrence, J. Doran, W.H. Colledge

**Affiliations:** a Department of Physiology, Development and Neuroscience, University of Cambridge, Cambridge CB2 3EG, United Kingdom; b Takeda Cambridge, 418 Science Park, Milton Road, Cambridge CB4 0PZ, United Kingdom

**Keywords:** Kisspeptin, Placental transport, *Kiss1*, *Gpr54/Kiss1r*, Knock-out mice

## Abstract

**Introduction:**

Kisspeptins, encoded by the *Kiss1* gene, are a set of related neuropeptides that are required for activation of the mammalian reproductive axis at puberty and to maintain fertility. In addition, kisspeptin signaling via the G-protein coupled receptor GPR54 (KISS1R) has been suggested to regulate human placental formation and correlations have been found between altered kisspeptin levels in the maternal blood and the development of pre-eclampsia.

**Methods:**

We have used *Kiss1* and *Gpr54* mutant mice to investigate the role of kisspeptin signaling in the structure and function of the mouse placenta.

**Results:**

Expression of *Kiss1* and *Gpr54* was confirmed in the mouse placenta but no differences in birth weight were found in mice that had been supported by a mutant placenta during fetal development. Stereological measurements found no differences between *Kiss1* mutant and wild-type placentas. Measurement of amino-acid and glucose transport across the *Kiss1* mutant placentas at E15.5 days did not reveal any functional defects.

**Discussion:**

These data indicate that mouse placentas can develop a normal structure and function without kisspeptin signaling and can support normal fetal development and growth.

## Highlights

• The structure and function of *Kiss1* and *Gpr54* mutant placentas was examined in transgenic mice.
• *Kiss1* and *Gpr54* mutant placentas are not associated with fetal loss during gestation or reduced pup weight at birth.
• *Kiss1* mutant placentas show normal structural components.
• *Kiss1* mutant placentas show normal transport of glucose and amino-acids.

## 1 Introduction

Kisspeptins, encoded by the *Kiss1* gene, are a set of overlapping neuropeptides that regulate the mammalian reproductive axis by binding to the G-protein coupled receptor GPR54 (also known as KISS1R) to stimulate GnRH secretion (for review see Ref. [1]. The primary protein product of 138 amino-acids in humans is cleaved to produce amidated peptides of different lengths including Kp54 (metastin), Kp14, Kp13 and Kp10 [2]. Kisspeptins were first purified from the human placenta, which has high levels of *KISS1* expression [2], [3]. In the human placenta, kisspeptin is expressed by syncytiotrophoblast cells [4], [5], [6] and kisspeptin levels in the maternal blood increase considerably from the eighth week of pregnancy until shortly after parturition [6], [7]. The kisspeptin receptor, GPR54, is also expressed by syncytiotrophoblasts, but also by villous and extravillous cytotrophoblast cells. Kisspeptins inhibit migration of the human extravillous trophoblast cell line HTR8SVneo [8] as well as primary cultures of human trophoblast cells [4], [9]. This reduced trophoblast migration may be caused by reduced expression of matrix metalloproteases [9] and/or increased adhesion to type I collagen in the extracellular matrix [10].

The inhibition of extravillous trophoblast migration has led to the suggestion that kisspeptins may have a role in regulating trophoblast invasion and placental development. Indeed, abnormal kisspeptin signaling in humans is associated with various pathologies of pregnancy, such as pre-eclampsia [11], [12], [13], [14], [15], [16] and impaired fetal growth [11], [17]. It is not clear, however, whether altered kisspeptin signaling is the primary cause of these pathologies, or a secondary effect of abnormal placental development. The aim of this study was to assess the expression of *Kiss1* and *Gpr54* in the murine placenta and analyze the structure and function of *Kiss1* mutant placentas to define any potential roles in mouse placentation. We used *Kiss1* mice rather than *Gpr54* mice for the structure/function studies to eliminate kisspeptin signaling through both the GPR54 receptor and neuropeptide FF receptors [18]. Although neuropeptide FF receptor expression has not been described in the mouse placenta, it has been shown in human placentas [19].

## 2 Methods

### 2.1 Mouse lines

All animals were 129S6/Sv/Ev mice with targeted disruption of *Gpr54* [20] or *Kiss1* [21]. The mice had ad-libitum access to food and water and a 12 h/12 h light/dark cycle (7am to 7pm). All procedures were performed under authority of a Home Office project and personal licence and approved by a local ethical review committee.

### 2.2 Timed matings

*Gpr54* or *Kiss1* heterozygous breeding pairs were set up to generate pregnant females that carried fetuses and placentas of all three genotypes (wild-type, heterozygotes and homozygous mutants). Females were checked for vaginal plugs each morning. On finding a plug the day was designated E0.5.

### 2.3 DNA isolation

#### 2.3.1 DNA isolation from ear clips

After weaning, a small ear biopsy was placed in 150 μl of alkaline lysis reagent (25 mM NaOH, 0.2 mM EDTA, pH 12). The sample was heated to 95 °C for 1 h and then cooled to 4 °C. 150 μl of neutralization buffer (40 mM Tris-HCL, pH 5.0) was added per tube. 1 μl of DNA was used per 25 μl PCR immediately or stored at −20 °C.

#### 2.3.2 DNA isolation from yolk sac/embryonic tissue

The mother was killed and the uterus dissected out. The gestational age of the embryos varied for each experiment and is stated in the methods and results sections for each experiment. Following dissection of the embryos, a small biopsy of either yolk sac or embryonic tissue was washed with sterile PBS to avoid contamination with maternal DNA before being placed in 100 μl of lysis buffer (50 mM KCl, 10 mM Tris–HCl (pH 8.3), 2 mM MgCl_2_, 0.1 mg/ml gelatin, 0.45% Tween-20, 0.45% Nonidet P40) and incubated at 55 °C overnight. The sample was then heated to 94 °C for 15 min and 1 μl was used per PCR reaction or stored at −20 °C.

### 2.4 Polymerase chain reaction (PCR) to analyze *Kiss1* and *Gpr54* expression

The PCR program used to genotype DNA from ear clips, yolk sacs and embryonic tissue was as follows: an initial 5 min at 95 °C followed by 44 cycles of 30 s at 93 °C, 1 min at 60 °C and 2 min at 70 °C. The primer sequences were *Kiss1*: Forward – tgctgcttctcctctgtgtcg; Reverse – gccgaaggagttccagttgta.

*Gpr54*: Forward – gccttcgcgctctacaacctgctg; Reverse – aaggcatagagcagcggattgagc.

Agarose gel electrophoresis was used to size PCR products with a Hyperladder IV DNA marker (Bioline, Cat no: Bio33029).

### 2.5 qRT-PCR analysis of *Gpr54* and *Kiss1* expression in E10.5 and E15.5 placentas

#### 2.5.1 Tissue collection

Mice were killed and the placentas dissected out at the appropriate time point of gestation. The placentas were rinsed in 1× PBS (pH 7.4) before being placed in RNAlater (Qiagen; 76104). They were stored at 4 °C overnight before being transferred to −80 °C.

#### 2.5.2 RNA extraction

RNA was extracted using RNeasy Plus Mini kit (Qiagen; 74134) according to the manufacturer's instructions. RNAase-Free DNAase (Qiagen; 79254) was used to ensure all genomic DNA had been removed. Gel electrophoresis was used to check the integrity of the samples. The concentration of the samples was measured using a NanoDrop 3300 spectrophotometer. Based on these results, equal concentrations of RNA were converted into cDNA using SuperScript III Reverse Transcriptase (Invitrogen; 18080 044) according to the manufacturer's instructions.

#### 2.5.3 Quantitative reverse transcriptase polymerase chain reaction using SYBR green

A SensiMix Plus SYBR kit (Quantace; QT605) was used to prepare the reaction mixture for quantitative PCR (qPCR). An ABI 7500 real-time thermal cycler was programmed as follows; 95 °C for 10 min followed by 40 cycles of 95 °C for 15 s, 60 °C for 15 s, 72 °C for 15 s. The sequences of the primers were: *Gapdh*: Forward – gatgcctgcttcaccacctcct; Reverse – aatgtgtccgtcgtggatctg.

*Gpr54*: Forward – tcactcggacccggatgtacaggtcag; Reverse – agcccgcgtacctgctggatgtagttg.

*Kiss1*: Forward – ccgtcatccagcctaagtttctcac; Reverse – ataggtggcgacacagaggagaagc.

A dissociation curve was added to confirm the presence of a single product. Serial dilutions (1, 1/5, 1/10, 1/50, 1/100) of cDNA were used with each primer pair. RNA that had not been converted to cDNA (minus RT sample) was used to identify genomic DNA contamination. Cycle threshold (Ct) values were plotted against log_2_ (template concentration) to analyze the efficiency of the primer pairs by calculating the gradient of each slope.

#### 2.5.4 Statistical analysis of qRT-PCR

The qRT-PCR data were analyzed using the 2^−ΔΔCt^ method [22].

### 2.6 Placental transfer assays

Heterozygous crosses were set up and monitored for copulatory vaginal plugs. The day of a plug was designated as E0.5. On E15.5 of pregnancy, unidirectional materno-fetal clearance of the non-metabolizable radioactive tracers ^14^C-methyl-isobutyric acid (MeAIB) and ^3^H-methyl d-glucose (MeG) were measured. Mice were anaesthetized with fentanyl-fluanisone (hypnorm): midazolam (hypnovel) in sterile water (1:1:2). A 100 μl bolus of 3.5 μCi of MeAIB (NEN NEC-671; specific activity 1.86 Gbq/mmol, Perkin Elmer, USA) or MeG (NEN NEC-377; specific activity 2.1 Gbq/mmol) in physiological saline (0.9% w/v) was injected intravenously into the maternal jugular vein. At 2 min after the tracer injection the mother was killed by cervical dislocation. Uteri were collected and the number of viable and resorbing implantation sites were counted and fetal and placental weights were recorded. Placental tissue was dissected into the Junctional and Labyrinthine zones and snap frozen in liquid nitrogen for qRT-PCR analysis. The yolk sac was taken for genotyping. All fetuses were minced for counting radioactivity.

Minced fetuses were lysed at 55 °C in Biosol (National Diagnostics, Atlanta, GA, USA) before measuring beta emissions of known aliquots by liquid scintillation counting (Optiphase Hisafe II, Perkin Elmer, USA; Packard Tri-carb, 1900, GMI Inc. USA). Fetally accumulated radioactivity was used to calculate placental clearance of MeAIB or MeG expressed as μl/min per gram of placenta. Counts accumulated in the fetus were expressed as total fetal disintegrations per minute (DPM) per gram fetus.

### 2.7 Placental stereology

Heterozygous crosses were set up and monitored for vaginal plugs. At E15.5 the female was killed and the uterus removed. Each fetus and its placenta was weighed. The placentas were hemisected using a razor blade, the weight of each half was recorded and they were then fixed immediately.

#### 2.7.1 Histology

The methods used here were based on Coan et al. [23]. Half of each placenta was fixed in 4% paraformaldehyde in 0.1 M PIPES buffer, dehydrated, and embedded in paraffin wax. A vibrotome was used to vertically section the entire placental halves into 7 μm thick sections. Systematic random sampling was used to select 10 sections for analysis. Sections to be analyzed were stained using a standard hematoxylin and eosin (H&E) protocol. The corresponding halves were fixed for 6 h with 4% glutaraldehyde in 0.1 M PIPES buffer, washed with 0.1 M PIPES buffer, and treated with 1% osmium tetroxide. The postfixed tissue was washed in 0.1 M PIPES buffer and dehydrated. This was followed by washes in propylene oxide, propylene oxide:Spurr resin (1:1), and Spurr resin:propylene oxide (2:1), then overnight flat embedding in 100% Spurr epoxy resin (Taab, Aldermaston, U.K.). Spurr resin was changed three times over 3 days, and the castings were thermally cured at 608 °C for 24 h. Sections, 1 μm thick, close to the placental midline were stained with Methylene blue.

#### 2.7.2 Analysis

The Computer Assisted Stereology Toolbox (CAST) 2.0 system from Olympus Ballerup, Denmark was used to perform all measurements.

#### 2.7.3 Absolute placental volume

To determine the absolute volume of placentas, a 32-point grid was superimposed on vertically orientated paraffin sections viewed using a 1.25× objective lens enabling a view of the complete sample. Points falling on the sample were counted and the Cavalieri principle applied to reach a volume estimate:

$$V_{\text{obj}}=t\sum a=ta_{\text{p}}\sum P$$

where *V*_obj_ is the estimated placental volume, *t* is the total thickness of the placenta (total number of sections multiplied by section thickness), *a*_p_ is the area associated with each point, and ∑*P* is the sum of points on sections.

#### 2.7.4 Volume of placental zones

Using the 10× objective lens, 12 fields of view on the sections used for determining the absolute placental volume were selected by meander sampling and measured by point counting to estimate component densities of the three zones (Lz, Jz, and db) using the equation:

$$V_{\text{v}\left(\text{struct},\text{ref}\right)}=P_{\left(\text{struct}\right)}/P_{\left(\text{total}\right)}$$

where *V*_v(struct,ref)_ is the volume fraction of a component (e.g., labyrinth zone) within a reference space (e.g., placenta), *P*_(struct)_ is the number of points falling on the component, and *P*_(total)_ is the total number of points falling on the reference space (including the component). The component volume densities obtained were converted to absolute quantities by multiplying by total placental volume.

#### 2.7.5 Labyrinth analyses

Resin sections were used to resolve the labyrinth vasculature in detail. A 100× objective lens was used and 12 fields of view within the labyrinth zone (Lz) were selected by meander sampling to determine volume densities.

The maternal blood space (MBS) and fetal blood space (FBS) were identified based on the appearance of the blood cells. Volume densities of the MBS, FBS, and trophoblasts were obtained using a point grid. Volume densities were converted to absolute component volumes by multiplying by the volume of the Lz.

### 2.8 Statistical analysis

Details of statistical tests applied to each data set are given in the figure legends. Statistical analyses of data were performed using InStat. *P* < 0.05 is considered significant.

## 3 Results

**Fig. 1:**
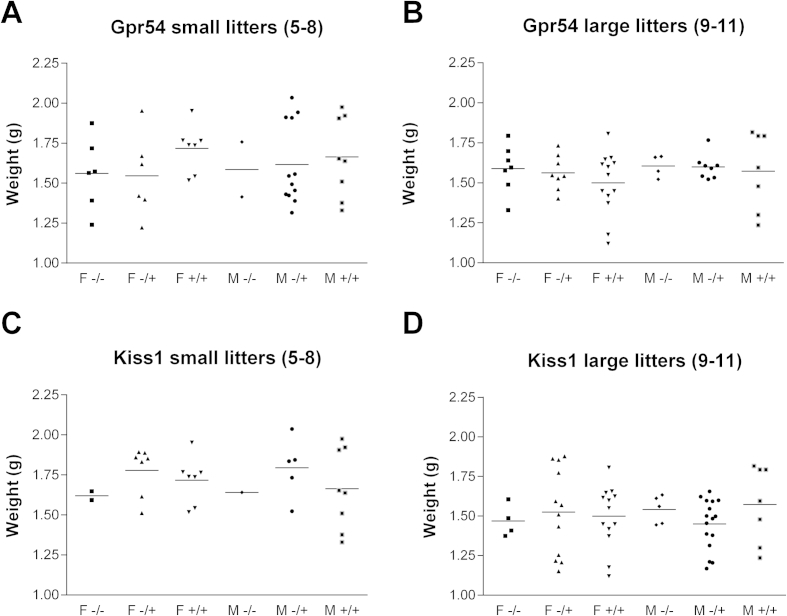
Weight of pups on day of birth compared with genotype and sex. A) Pups from *Gpr54* large litters (*n* = 47). B) Pups from *Gpr54* small litters (*n* = 41). C) Pups from *Kiss1* large litters (*n* = 57) D) Pups from *Kiss1* small litters (*n* = 30). Mean values are shown. F, female; M, male. There was no statistically significant difference between any of the categories (One-way ANOVA).

The birth weight of pups can provide an indicator of placental function during gestation [24]. The birth weights of *Gpr54* and *Kiss1* mutant mice were compared to wild-type and heterozygous littermates to assess whether mutant placentas compromised fetal growth. As litter size is known to affect the birth weight of individual animals, we separated the pups into small (5–8 pups) and large (9–11 pups) litter groups as well as by sex (Fig. 1). The genotype of the pups had no significant effect on the average birth weight suggesting that the absence of kisspeptin signaling in the mouse placenta is not associated with compromised fetal growth.

**Fig. 2:**
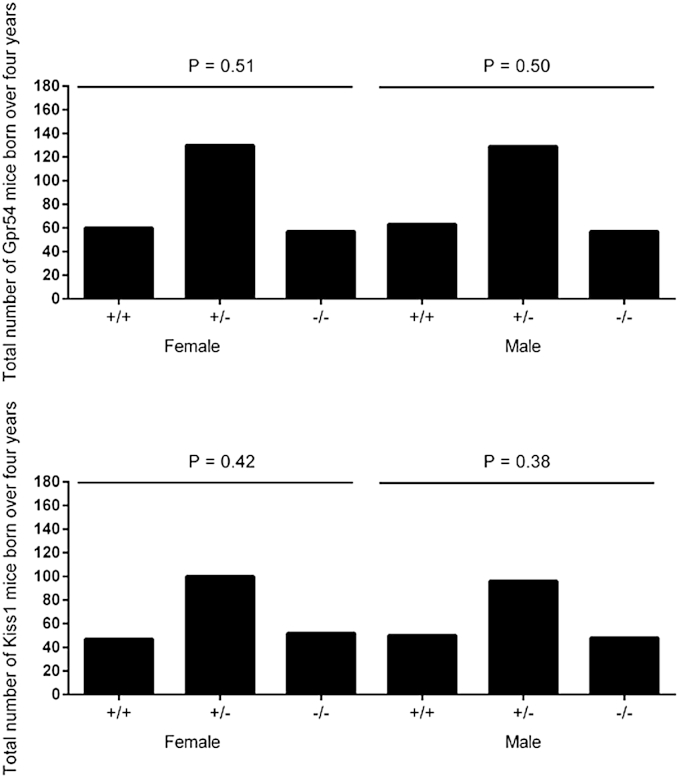
Analysis of the number of mice of each genotype born over a 4 year period. *P* values were calculated using a Chi-squared test of the expected Mendelian frequencies of all genotypes. There was no significant deviation from the expected numbers for each genotype.

In humans, decreased kisspeptin expression in trophoblast cells during the first trimester has been linked to recurrent pregnancy loss [25], therefore birth rates were analyzed over a four year period to see if there was evidence of increased *in utero* loss of mutant mice (Fig. 2). Inheritance patterns conformed to the expected Mendelian inheritance ratios and no difference was found between the *Kiss1* and the *Gpr54* mutant lines.

**Fig. 3:**
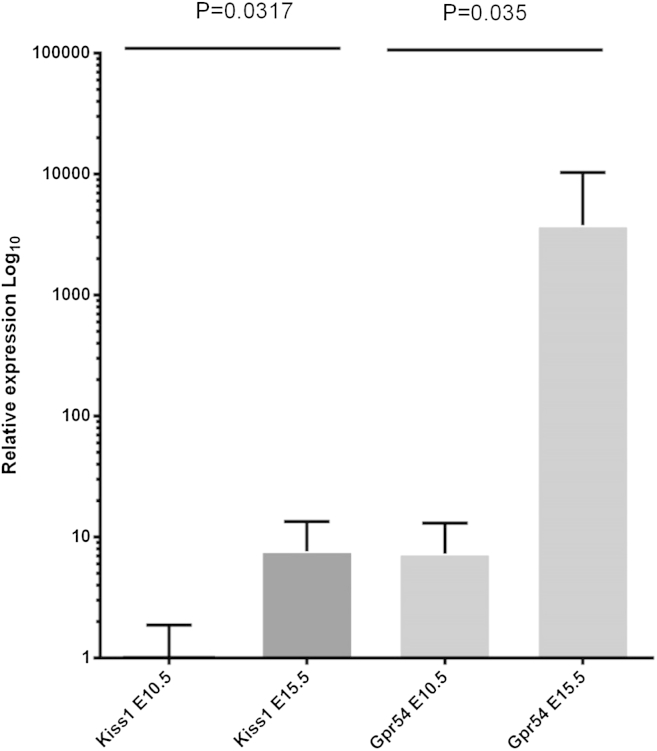
Expression analysis of *Kiss1* and *Gpr54* transcripts in the mouse placenta. Transcript levels were measured by qRT-PCR at E10.5 and E15.5. There was a significant rise in both *Gpr54* and *Kiss1* expression between E10.5 (*n* = 5) and E15.5 (*n* = 5). *P* values are shown (*t*-test).

Expression of *Gpr54* and *Kiss1* was examined in wild-type placentas at E10.5 and E15.5 by quantitative RT-PCR (Fig. 3). The data show that both *Gpr54* and *Kiss1* are expressed in the mouse placenta but *Kiss1* mRNA levels are very low. Expression of both *Gpr54* and *Kiss1* increased between E10.5 and E15.5 and expression of *Gpr54* was always higher than *Kiss1* (Fig. 3).

**Fig. 4:**
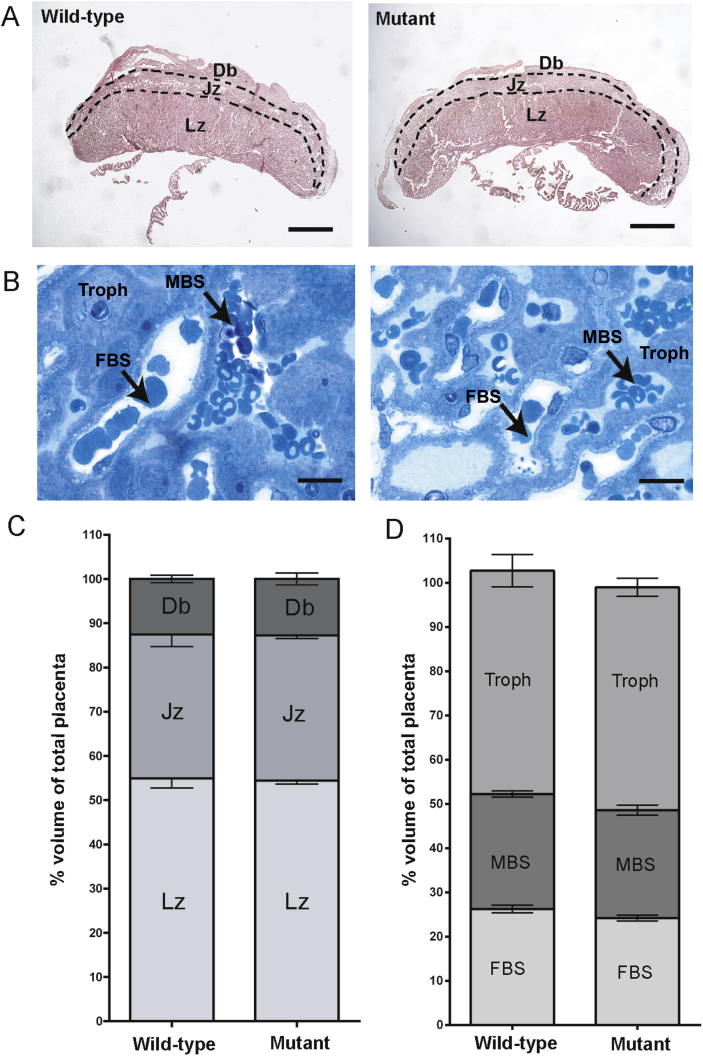
Structural analysis of *Kiss1* mutant placentas at E15.5. A) Low power photomicrographs of placentas illustrating the different zones. Lz = Labyrinth zone, Jz = junctional zone, Db = decidua basalis. Scale bar = 1 mm. B) Identification of components of the labyrinth vasculature. Fetal blood space (FBS), maternal blood space (MBS) and trophoblast cells (Troph) were identified in the labyrinth zone. Scale bar = 10 μM. C) Stereological measurement of the sizes of the placental zones in *Kiss1* mutant placentas. 6 wild-type and 5 *Kiss1* mutant placentas were analyzed. Bars indicate ± SEM. *P* > 0.05 (*t*-test). D) Comparison of the percentage volume of the labyrinth zone components. 6 wild-type and 5 *Kiss1* mutant placentas were analyzed using stereological methods. Bars indicate ± SEM. *P* > 0.05 (*t*-test).

Stereological analysis of the placentas was performed to investigate if there were any alterations in the size of each placental zone. It was found that there were no differences in the volumes of the different placental zones between wild-type and *Kiss1* mutant mice (Fig. 4A). Specifically, the volume of the labyrinth zone was not altered in the *Kiss1* mutant mice (Fig. 4C). A more detailed stereological analysis of the labyrinth zone in *Kiss1* mutant placentas was performed. The area of trophoblast cells, and fetal and maternal blood spaces was measured (Fig. 4B). No significant differences were found in the major structural components of the labyrinth zone (Fig. 4D).

**Fig. 5:**
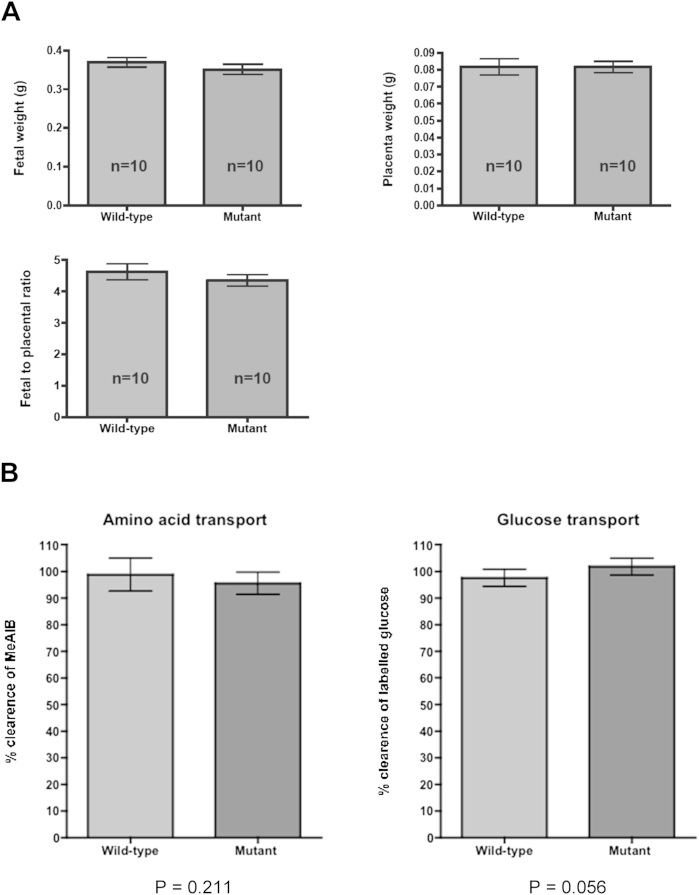
Functional analysis of *Kiss1* mutant placentas at E15.5. A) Fetal and Placental weight parameters. Comparisons between wild-type and *Kiss1* mutant fetuses and placentas. 10 fetuses were analyzed in each category. All *P* > 0.05 (*t*-test). B) Relative amino acid and glucose transport across the placenta. At E15.5 of pregnancy unidirectional materno-fetal clearance of the non-metabolizable radioactive tracers ^14^C-methyl-isobutyric acid (MeAIB) and ^3^H-methyl d-glucose (MeG) were measured. Fetally accumulated radioactivity was used to calculate placental clearance of MeAIB and MeG. Placentas from 7 wild-type and 6 *Kiss1* mutant mice were analyzed. Bars indicate ± SEM. *P* > 0.05 (*t*-test).

Fetal and placental weights and the ratio were measured as an indicator of placental function and this showed that there was no significant difference between *Kiss1* mutants and wild-type mice (Fig. 5A). The stereological data suggest that nutrient transfer across a mutant placenta should not be compromised but we performed a functional test of amino-acid and glucose transport to confirm this. No alteration in the transport capacity of *Kiss1* mutant placentas was found (Fig. 5B).

## 4 Discussion

We have examined *Kiss1* mutant placentas in mice to establish whether absence of kisspeptin signaling alters placental structure or function. Although we detected both *Gpr54* and *Kiss1* expression in the mouse placenta at E10.5 and E15.5, the level of *Kiss1* transcripts was very low. We did not find any differences in the *Kiss1* mutant placentas in the size of any of the placental zones or in the cellular components of the labyrinth zone, which is the major region responsible for nutrient transfer across the placenta. Indeed, measurement of amino-acid and glucose transport across *Kiss1* mutant placentas did not reveal any defects which was consistent with no reduction in the birth weight of the mutant pups. This data suggests that kisspeptin signaling does not play a major role in placental function in the mouse. One caveat however, is that since we used heterozygous females in these studies, our experiments were not designed to identify any maternal factors that might influence placental function such as uterine artery formation.

Our quantification of *Kiss1* and *Gpr54* expression indicated that *Kiss1* transcripts were lower than *Gpr54* in placentas at both E10.5 and E15.5 of gestation with *Gpr54* transcripts at E15.5 being 300× greater than *Kiss1* (Fig 3). This is in contrast to humans where *Kiss1* mRNA is usually higher than *Gpr54* [12], [26], [27] and in the rat, where *Kiss1* transcripts are around 20× greater than *Gpr54* [28], [29]. The reason why *Gpr54* transcripts were higher than *Kiss1* in the mouse is probably related to the extremely low expression of *Kiss1* mRNA in this species compared to other species. In the human placenta, *KISS1* is highly expressed at around 17% relative to GAPDH transcript levels which equates to 15,000 mRNA copies/ng mRNA [27]. In the rat placenta, *Kiss1* expression is 20 mRNA copies/ng mRNA [29] which represents around 0.02% of *Gapdh* expression which is still sufficient to be higher than *Gpr54*. We estimate that in the E15.5 mouse placenta, *Kiss1* mRNA expression is around 0.000003% compared to *Gapdh*, which makes the relative level of *Gpr54* seem very high. Consistent with this extremely low level of *Kiss1* mRNA expression, we have failed to detect either *LacZ* expression from the targeted allele [21] or kisspeptin protein by immunohistochemistry in mice (data not shown). Moreover, measurement of kisspeptin protein in rat placentas shows a very low level of expression (1.26 fmol/mg tissue) [30]. Clearly *Kiss1* expression is considerably higher in human placentas than in rodents. Indeed, elevated kisspeptin levels in the maternal blood analogous to those found in pregnant women have not been reported in mice.

Our data suggest that kisspeptin signaling does not have a significant role in mouse placental structure or function. It is possible, however, that the mutant placentas may be rescued by kisspeptin production from heterozygous and wild-type littermates present in the same uterus. We think that this is unlikely however given the similar birth weights of the *Kiss1* and *Gpr54* mutant mice. If absence of kisspeptin signaling in the *Kiss1* mutant placentas was being rescued by kisspeptins produced by other placentas then we might expect to see a difference specifically in the birth weights of the *Gpr54* mutant pups. A comparison of the birth weight of all of the *Gpr54* mutant pups of both sexes (*n* = 21) with wild-type pups (*n* = 20) indicated no significant difference between the average birth weights. We are also not aware of any situation where the phenotype of a mutant fetus has been rescued by trans-placental movement of a secreted protein. For example, paternal transmission of a mutated *Igf-II* allele which causes growth deficiency in heterozygous placentas and fetuses is not prevented by IGF-II secretion from adjacent wild-type placentas [31], [32]. Moreover, our qRT-PCR analysis indicates that the expression level of the *Kiss1* gene in the mouse placenta is so low as to be of questionable physiological significance.

Although our data indicate that kisspeptin signaling is not required for normal placentation in the mouse, it is likely that kisspeptins will have additional roles in the mouse female reproductive tract. For example, Calder et al. [33] have shown that blastocyst implantation in *Kiss1* null mice does not occur unless the mice are injected with exogenous leukemia inhibitory factor (LIF) suggesting that uterine kisspeptin signaling regulates LIF production. The ability of embryo implantation to occur in *Gpr54* null mice where fertility has been rescued by restricted expression of a *Gpr54* transgene in GnRH neurons [34] suggests that the kisspeptin induction of LIF may be mediated by NPFF receptors rather than GPR54. Alternatively, the *Gpr54* transgene might be expressed in the uterine epithelium of these rescued mice but this was not examined by the authors. Kisspeptin signaling may also play a role in oogenesis as *Kiss1* and *Gpr54* expression have been found in ovarian tissue from rat, hamster and primates [35], [36], [37]. Recently, *Gpr54* heterozygous female mice have been reported to show age-related loss of ovarian follicles and develop premature ovarian failure [38]. These studies indicate that kisspeptin signaling may have roles in ovulation, implantation and pregnancy and further work will be needed to define these as well as identify possible species differences.

In women, placentally derived kisspeptin levels increase around the eighth week of pregnancy and remain elevated throughout gestation [6], [7]. Reduced maternal serum kisspeptin levels between eight and fourteen weeks of gestation are associated with small-for-gestational age neonates [17]. It has also been reported that decreased maternal serum kisspeptin levels in the second trimester are associated with interuterine growth retardation [11]. Correlations have also been observed between low maternal kisspeptin levels during pregnancy and pre-eclampsia in humans [11], [12], [13], [14], [15], [16]. It is possible, therefore, that kisspeptin may have a role in human placental function but this is not required in the mouse. It should be noted however, that live individuals with mutations in *GPR54* indicates that kisspeptin signaling is not essential for the fetal part of the placenta to form in humans. Moreover, the ability of women with mutations in *GPR54* to receive hormone treatments and maintain a pregnancy to term shows that uterine expression of *GPR54* is also not essential for pregnancy [39]. What is not yet know is whether absence of GPR54 expression in both the uterus and the fetus is compatible with normal placentation and fetal growth.

It is also possible however, that the reduced maternal kisspeptin levels associated with fetal growth retardation or pre-eclampsia in humans are secondary to a defect in placenta formation rather than the primary cause. If this is the case, then kisspeptins would not responsible for placental dysfunction in humans but could still be used as a useful biomarker of placental function.

So far, the human data suggest a link between kisspeptin signaling and placentation whereas our mouse data show that kisspeptin signaling does not seem to play a major role in placentation. Further investigations in humans are required to understand if altered kisspeptin signaling has a direct role in placentation or if altered kisspeptin signaling is a secondary effect of placental dysfunction. Based on our findings, it is unlikely that the mouse will be a suitable model to investigate this further.

## Conflict of interest

I declare that there are no conflicts of interest with regard to this manuscript.

## Disclosure statement

The authors have nothing to disclose.

## Grants and fellowships

This work was supported by the BBSRC (Grant number BB/F01936X/1), Alice Herreboudt was supported by a BBSRC CASE studentship in collaboration with Takeda Cambridge.
